# School bullying int Tunisia: Psychological profile of harassers

**DOI:** 10.1192/j.eurpsy.2021.558

**Published:** 2021-08-13

**Authors:** Z. Azouz, T. Brahim, N. Ben Salem, R. Ayoub, N. Brigui, I. Ben Turkia, A. Guedria

**Affiliations:** 1 Child And Adolescent Psychiatry, Fattouma BOURGUIBA Hospital, Monastir, Tunisia; 2 Child And Adolescent Psychiatry Department, Fattouma Bourguiba University Hospital, Monastir, Tunisia, Monastir, Tunisia; 3 Child And Adolescent Psychiatry, Fattouma bourguiba University Hospital, Monastir, Tunisia

**Keywords:** adolescent, bullying, Profile, Harasser

## Abstract

**Introduction:**

School Bullying is an educational and a health care issue that was kept hided for a long time. Despite, growing interest about this issue, it’s still a topic that is not well known and well analysed in Tunisia.

**Objectives:**

- Evaluate the prevalence of school bullying among Middle school students - Establish the psychological profile of harassers

**Methods:**

It’s a cross sectional study including 600 students of 2 middle schools of the region of Sousse -Tunisia during the month of March 2020. “The adolescent peer relations instrument” was used to identify school bullying and its type. The “Child Behaviour Checklist” questionnaire was used to identify emotional and behavioural problems among children. Self esteem levels were evaluated by the “Rosenberg Self Esteem Scale”.

**Results:**

The mean age of participants was 13 years and 9 months ±1 year and 4 months. 95.1 % of the participants have experienced school harassment, but also 92% of them harassed their peers. Boys were more frequently bullies than girls (p<0.01). There was no significant difference in self esteem levels between bullies and non-bullies adolescents. Among the 8 syndroms explored by the Child behaviour checklist, adolescents experiencing one of the 7 folllowing items (Anxious syndrome, Depressive syndrome, Aggressive behaviour, Attention problems, Social problem, Thoughts problems and Withdrawal problems) had significantly higher risk of being a bully (p values between 0.001 and 0.02).

**Conclusions:**

This study emphasizes the high prevalence of school bullying among adolescents in Tunisia. Most of the psychological problems explored in this study seem to be higher among bullies.

